# Supplementary material for: Strain-tunable van der Waals interactions in few-layer black phosphorus
Source: Nat Commun. 2019 Jun 4;10:2447. doi: 10.1038/s41467-019-10483-8 (PMC6547657; doi:10.1038/s41467-019-10483-8)
Supplement: Supplementary file 1 — Supplementary Information [file 41467_2019_10483_MOESM1_ESM.pdf]

Supplementary Information for

Strain-tunable van der Waals interactions in few-layer black phosphorus

Shenyang Huang. et al.

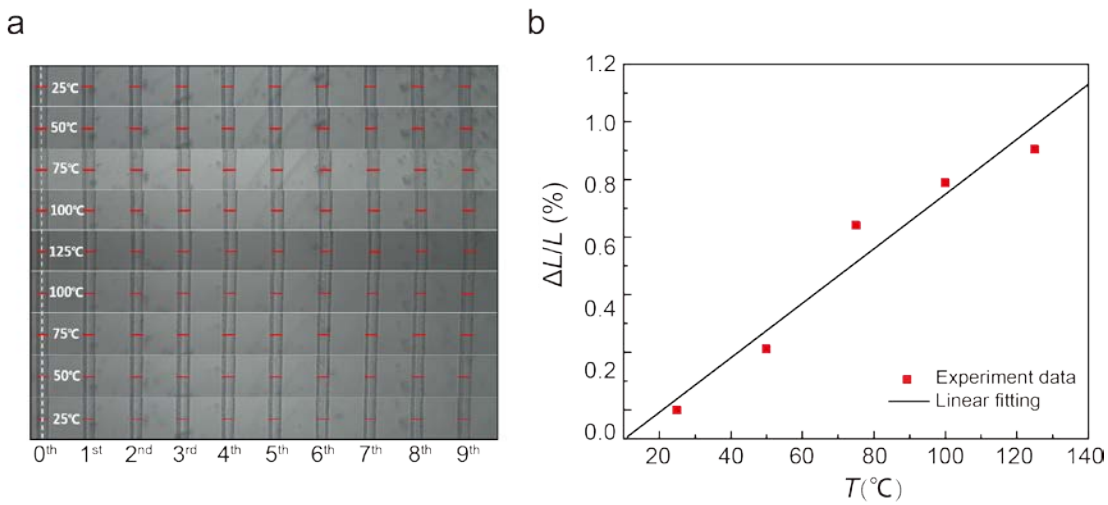

Supplementary Figure 1 | Thermal expansion coefficient of PP substrate. (a) Optical images of a polypropylene (PP) substrate with periodic trenches at different temperatures (25-125 °C). (b) The relative change ($\Delta L/L$) of the spacing between the 0th and 9th trenches as a function of temperature is plotted, with the data averaged from heating and cooling processes. A linear relation was observed in our temperature range. The thermal expansion coefficient of PP is determined as 0.9×10^{-4}/°C from the slope of the linear fitting. The strain value can be determined through the formula $\varepsilon=\alpha(T - T_0)$, where T is the temperature and T_0 corresponds to the temperature at zero strain.

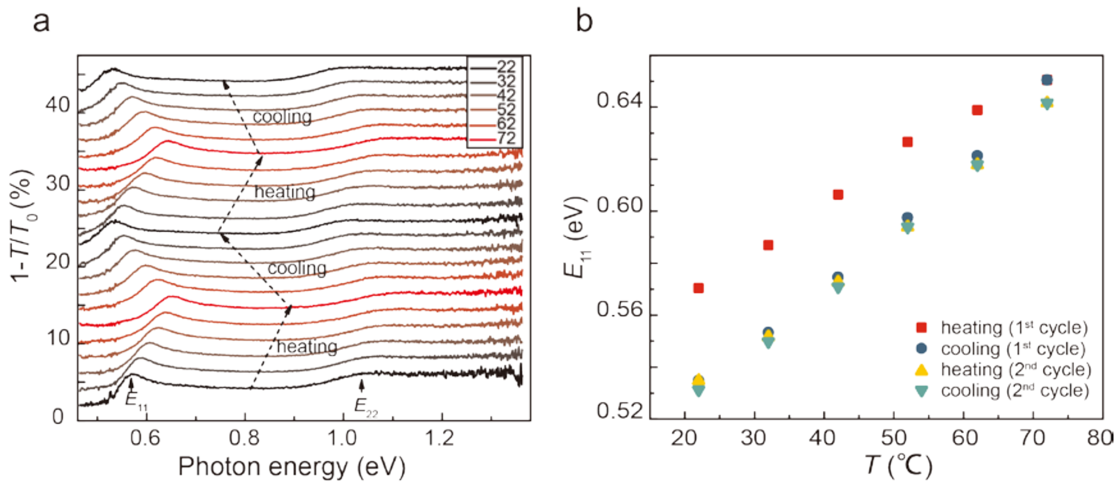

Supplementary Figure 2 | Repeated biaxial strain during multiple heating and cooling cycles. (a) Extinction spectra for a 6L BP on PP substrate upon two heating and cooling cycles. To avoid possible slippage between BP flakes and PP substrates as well as non-uniform deformation of PP, the temperature variation was kept within a small range of 20-75℃ (corresponding to the biaxial strain range of -0.3 - 0.3%) (b) E_{11} transition energy of the 6L BP as a function of temperature. As it shows, except for the first heating process, the repeatability is very good during all of the subsequent cooling and heating cycles. This is because after the first heating process, the temperature corresponding to zero strain (T_0) is no longer the initial room temperature. The adhesiveness between BP flakes and PP substrates is enhanced after heating. Consequently, as the temperature cools back to the room temperature, the BP flake is actually under compressive strain. Due to different initial mechanical states for different samples, the zero-strain temperature (T_0) differs from sample to sample, but it (T_0 typically around 50 °C) does not vary substantially. Since we are only interested in the slope of transition energy as a function of temperature, the value of T_0 doesn't affect peak shift rates.

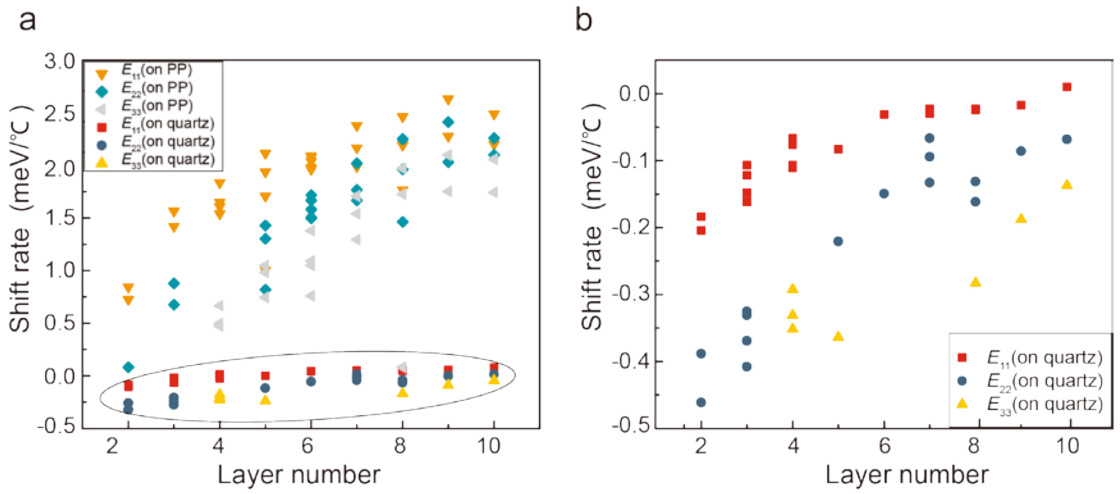

Supplementary Figure 3 | Pure temperature effect for few-layer BP (a) Comparison of the shift rates (cm^{-1}/°C) of E_{nn} transition energies of 2-10L BP on PP and quartz substrates. Compared with PP, quartz has a much smaller thermal expansion coefficient ($\alpha_{quartz} \approx 0.04\times10^{-4}$/°C) and a better thermal conductivity[1]. Thus, quartz substrate is quite suitable for us to differentiate the temperature effect from the strain effect. For BP on quartz substrate, the shift rates are mainly originated from the pure temperature effect and are almost negligible compared to the PP case. In view of this, it is obviously that for BP on PP substrate, the strain effect overwhelmingly dominates over the pure temperature effect, which largely simplifies our analysis. (b) A zoom-in of the data in the black circle in Fig. 3a.

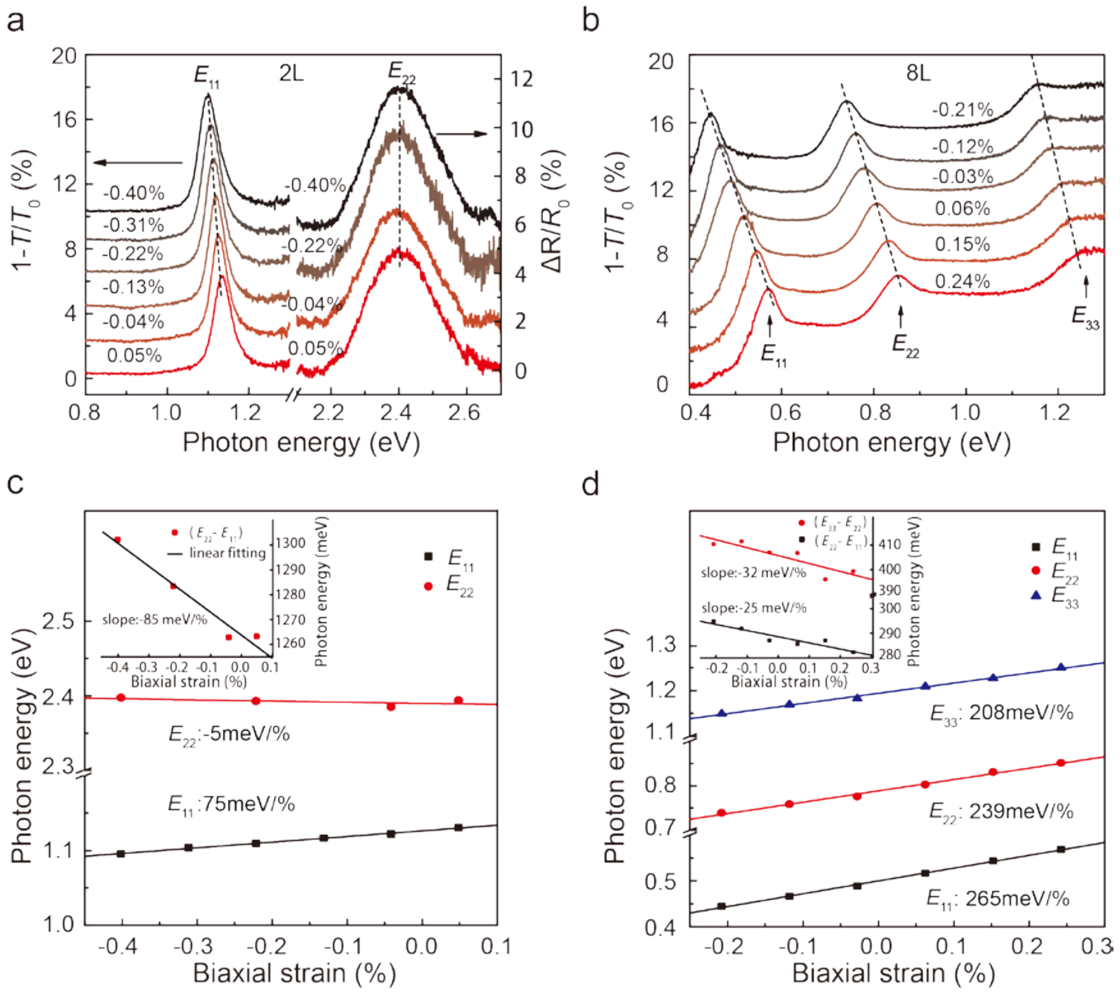

Supplementary Figure 4 | Subband index-dependent biaxial strain effect. IR extinction spectra of strained (a) 2L and (b) 8L BP. For 2L BP, E_{22} transition energy lies in the visible range, beyond the measurement range of our FTIR, thus we used another setup (a grating spectrometer combined with a microscope) to complement the IR range. (c) and (d) Optical transition energies of 2L and 8L BP as a function of biaxial strain. Dots are experiments data and solid lines are linear fits. The shift rates of 2L E_{11} and E_{22} are 75 and -5 meV/% respectively. For 8L E_{11}, E_{22} and E_{33} transitions, shift rates are 265, 239 and 208 meV/%, respectively. The inserts in Figs. 4c, d show (E_{22}-E_{11}) in 2L and (E_{22}-E_{11}), (E_{33}-E_{22}) in 8L as a function of biaxial strain respectively.

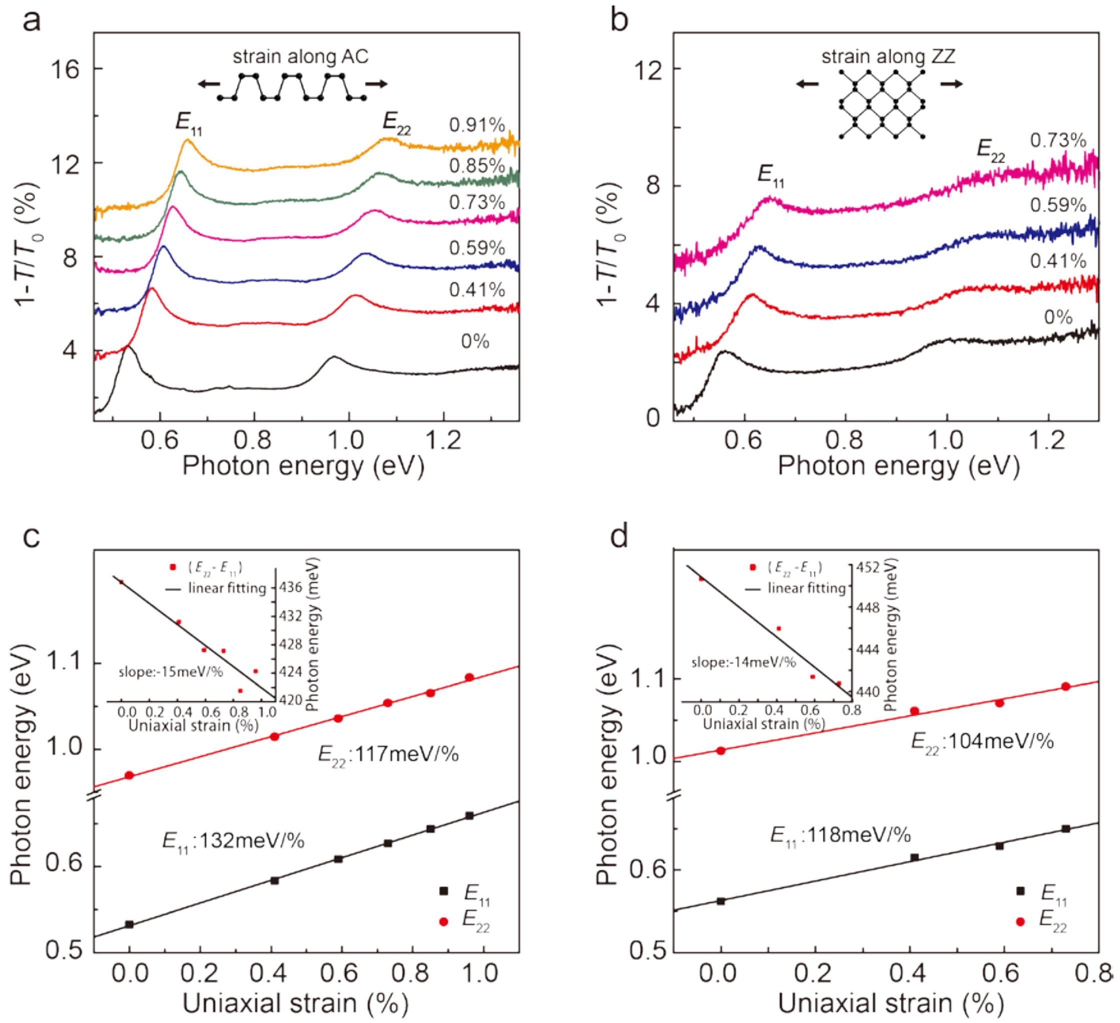

Supplementary Figure 5 | Subband index-dependent uniaxial strain effect. IR extinction spectra of a 6L BP under uniaxial tensile strain along (a) AC and (b) ZZ directions. Data shown in (a) and (b) come from two different 6L BPs. (c) and (d) Optical transition energies of a 6L BP as functions of tensile strain along AC and ZZ, respectively. Dots are experimental data and solid lines are linear fits. The shift rates of E_{11} and E_{22} are (132, 117), (118,104) meV/%, with strain along AC and ZZ directions, respectively. The inserts in Figs. 5c, d show the energy difference (E_{22}-E_{11}) as a function of uniaxial strain along AC and ZZ directions, respectively. The experimental details for applying in-plane uniaxial strain can be found in a previous

publication[2]

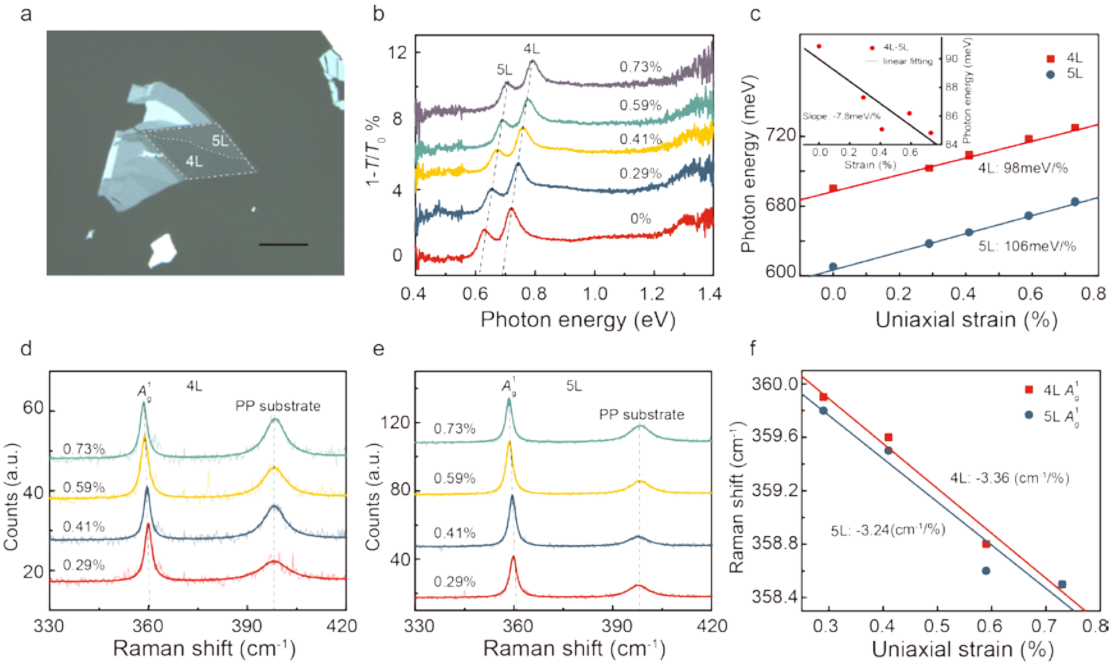

Supplementary Figure 6 | Layer-dependent uniaxial strain effect (strain along AC). (a) Optical image of the adjacent 4L and 5L BP flakes. Scale bar: 40 μm. (b) IR extinction spectra for the 4L and 5L BP under different uniaxial strains along AC direction. (c) Transition energies of E_{11} for the 4L and 5L BP as functions of uniaxial strain. The solid lines are linear fits to the data. The insert shows E_{11} energy difference of 4L and 5L BP as a function of uniaxial strain. The shift rates of E_{11} are 98 and 106 meV/% for 4L and 5L, respectively, which are about half of those induced by biaxial strain. (d) and (e) Raman spectra for the 4L and 5L BP under different uniaxial strains along AC direction, respectively. As discussed in the main text, here we also choose the A_g^1 mode as an indicator for the strain magnitude. (f) Peak positions of A_g^1 mode of 4L and 5L BP as functions of uniaxial strain. The solid lines are linear fits.

The almost equal shift rates suggest the same strain condition.

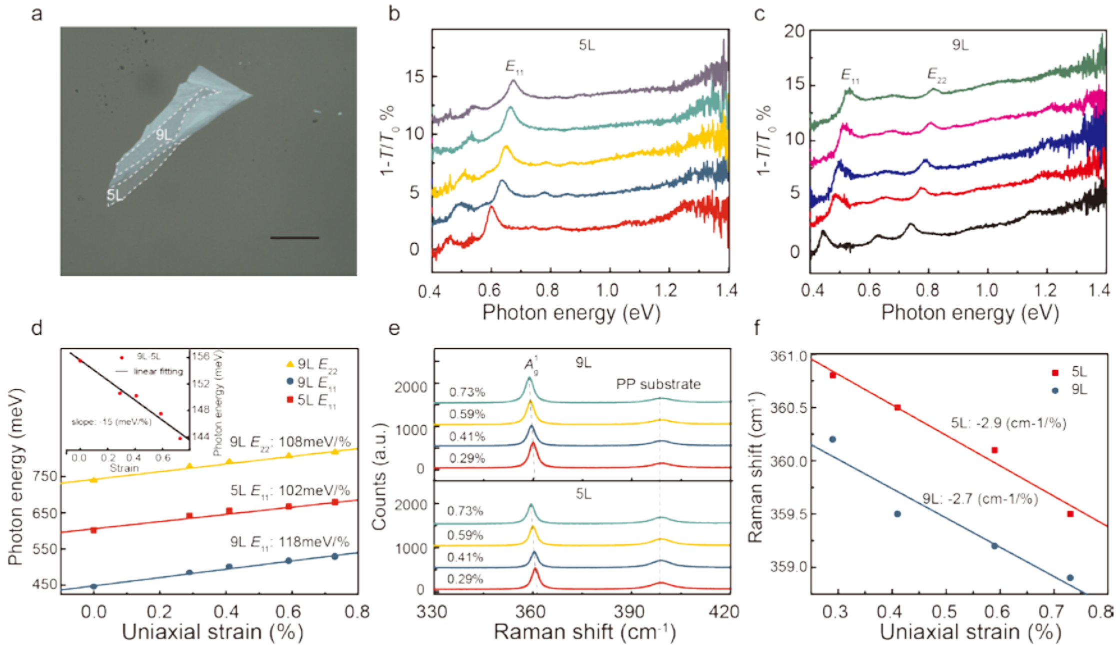

Supplementary Figure 7 | Layer-dependent uniaxial strain effect (strain along ZZ). (a) Optical image of the adjacent 5L and 9L BP flakes. Scale bar: 40 µm. (b) and (c) IR extinction spectra for the 5L and 9L BP under different uniaxial strains along ZZ direction, respectively. (d) Transition energies of E_{11} of the 5L and E_{11}, E_{22} of 9L BP as functions of uniaxial strain. The solid lines are linear fits to the data. The insert shows E_{11} energy difference of 5L and 9L BP as a function of uniaxial strain. The shift rates of E_{11} are 102 and 118 meV/% for 5L and 9L, respectively. (e) Raman spectra for the 5L and 9L BP under different uniaxial strains along ZZ direction, respectively. (f) Peak positions of A_g^1 mode of 4L and 9L BP as functions of uniaxial strain. The solid lines are linear fits. The very similar shift rates suggest the same strain condition.

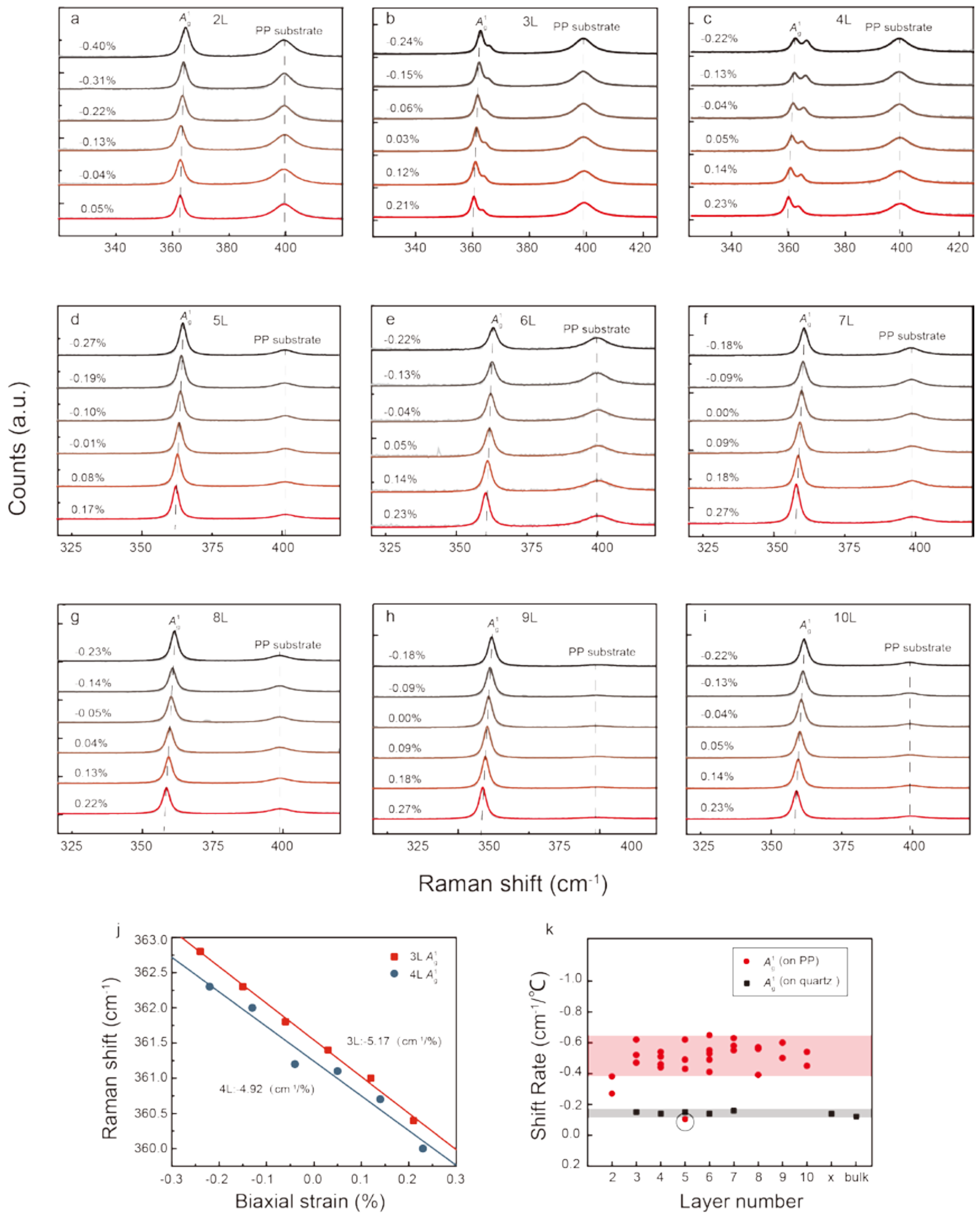

Supplementary Figure 8 | Raman spectra of strained few-layer BP. (a)-(i) Raman spectra for few-layer BP under different strains with layer number *N*=2-10. Dashed lines are guides to the eye. According to our discussion in the main text, we choose

the A_g^1 mode as an indicator for the strain magnitude. (j) Peak positions of A_g^1 mode of 3L and 4L BP (shown in Fig. 2a of the main text) as functions of biaxial strain. The solid lines are linear fits. The very similar shift rates suggest that these samples are under the same strain condition. (k) Shift rates of A_g^1 mode as functions of layer number on two different substrates. The layer number *x* is estimated to be 15-20. As mentioned above, the shift rates are mainly originated from the pure temperature effect on quartz substrate, which is ~0.015 cm^{-1}/°C, in good agreement with previous studies[3] and almost layer-independent, which assures the validity of our assumption in the main text. While on PP substrates, the shift rates are mainly originated from the strain effect. As shown in the red area, similar shift rates guarantee that these samples are under similar strain condition. If there is obvious slippage between BP flake and PP substrate, the shift rate will be much lower than the normal value and hence the corresponding shift rate of optical resonance E_{nn} will be abnormally low (e.g, the shift rate of a 5L sample shown in black cycle is abnormally low and the corresponding shift rates of E_{11}, E_{22} are only 109 meV/% and 87 meV/%, which is almost half of the normal value, suggesting a serious slippage between this 5L flake and substrate. Thus such samples will be excluded from our database of strain results.)

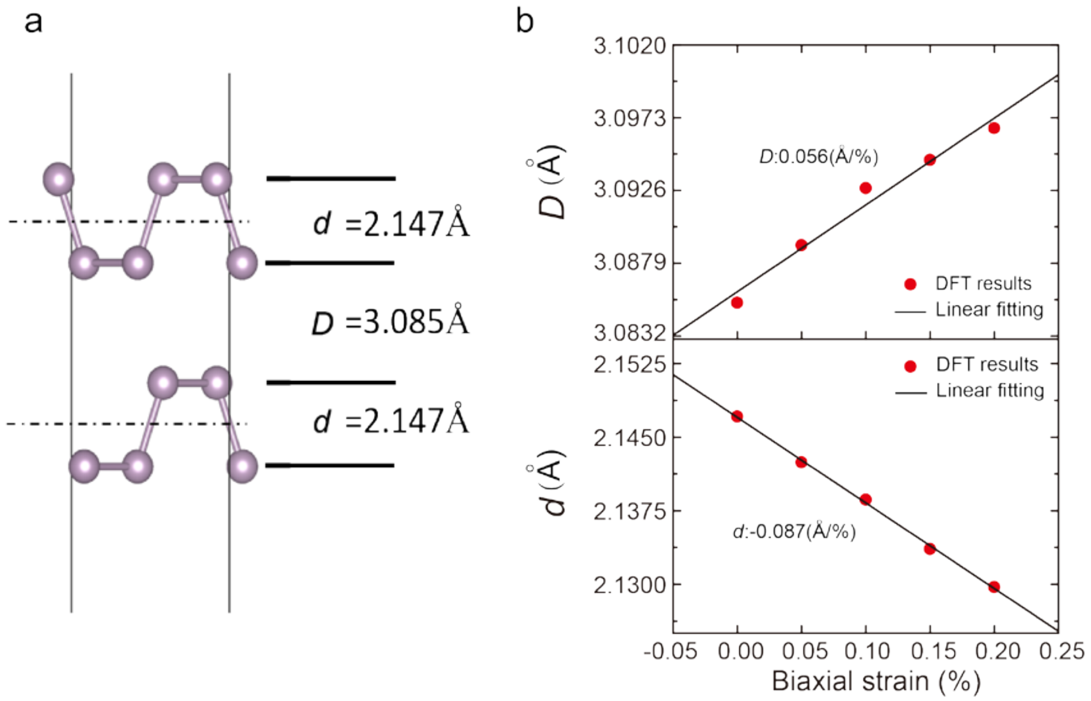

Supplementary Figure 9 | Layer and sub-layer distances under biaxial in-plane strain in 2L BP calculated by DFT (a) Side view of the unstrained 2L BP: *d* is the distance between two sub-layers within a layer and *D* is the gap between two layers. The average distance between two phosphorene layers is thus *D*+*d*. (b) *D* and *d* versus biaxial tensile strain, calculated by DFT. When 1% biaxial tensile strain is applied, sub-layer distance *d* decreases by 0.087 Å, while the gap *D* between two adjacent layers increases by 0.056 Å. Thus, interlayer distance (*D*+*d*) between two phosphorene layers decreases by 0.031 Å, corresponding to an out-of-plane Poisson's ratio $\nu = -\frac{d\varepsilon_z}{d\varepsilon_{xy}}$ of ~0.5, very similar to previous DFT results (0.57) (Ref.[4]). In the expression, ε_{xy} and ε_z are the in-plane and out-of-plane strains, respectively.

Supplementary Note |Quasi-1D tight binding model

The transition energy E_{nn} at Γ point of the 2D Brillouin zone of a N-layer BP is expressed as[2]:

$$E_{nn} = E_g - 2(\gamma_c - \gamma_v)\cos(\frac{n\pi}{N+1}) \\ = E_g - 2\Delta\gamma\cos(\frac{n\pi}{N+1}) \tag{1}$$

where E_g is the bandgap of mono-layer BP, γ_c, γ_v are the overlapping integrals of conduction and valence bands, respectively, which are proportional to the interlayer hopping parameter ($t_\perp$), n is the subband index. We assume that E_g and $\Delta\gamma$ ($\gamma_c - \gamma_v$) change linearly with biaxial strain:

$$E_g = E_{g0} + h\varepsilon \qquad \Delta\gamma = \Delta\gamma_0 + k\varepsilon \tag{2}$$

where ε is strain and h, k are coefficients, E_{g0} and $\Delta\gamma_0$ are E_g and $\Delta\gamma$ under zero strain respectively (From our previous study[2], we have E_{g0}=2.12eV, $\Delta\gamma_0$=0.88eV). Then we have:

$$E_{nn} = A_n + h\varepsilon - 2k\varepsilon\cos(\frac{n\pi}{N+1}) \tag{3}$$

where $A_n = E_{g0} - 2\Delta\gamma_0\cos(\frac{n\pi}{N+1})$ is the zero strain transition energies. Thus, the shift rate of E_{nn} is:

$$\frac{dE_{nn}}{d\varepsilon} = h - 2k\cos(\frac{n\pi}{N+1}) \tag{4}$$

From equation (2), we see that a negative k value means a decrease of interlayer coupling under tensile strain.

Supplementary References

1 Frisenda, R. *et al.* Biaxial strain tuning of the optical properties of single-layer transition metal dichalcogenides. *Npj 2d Mater Appl* **1**, 10 (2017).

2 Zhang, G. *et al.* Infrared fingerprints of few-layer black phosphorus. *Nat Commun* **8**, 14071 (2017).

3 Łapińska, A., Taube, A., Judek, J. & Zdrojek, M. Temperature Evolution of Phonon Properties in Few-Layer Black Phosphorus. *The Journal of Physical Chemistry C* **120**, 5265-5270 (2016).

4 Hu, T., Han, Y. & Dong, J. Mechanical and electronic properties of monolayer and bilayer phosphorene under uniaxial and isotropic strains. *Nanotechnology* **25**, 455703 (2014).
